# Bidirectional association between blood pressure and depressive symptoms in young and middle-age adults: A cohort study

**DOI:** 10.1017/S2045796020000542

**Published:** 2020-07-15

**Authors:** Sang Won Jeon, Yoosoo Chang, Se-Won Lim, Juhee Cho, Han-Na Kim, Kyoung-Beom Kim, Jinseok Kim, Young Hwan Kim, Dong-Won Shin, Kang-Seob Oh, Young-Chul Shin, Seungho Ryu

**Affiliations:** 1 Department of Psychiatry, Kangbuk Samsung Hospital, Sungkyunkwan University School of Medicine, Seoul, Republic of Korea; 2 Center for Cohort Study, Total Healthcare Center, Kangbuk Samsung Hospital, Seoul, Republic of Korea; 3 Department of Occupational and Environmental Medicine, Kangbuk Samsung Hospital, Sungkyunkwan University School of Medicine, Seoul, Republic of Korea; 4 Department of Clinical Research Design & Evaluation, SAIHST, Sungkyunkwan University, Seoul, Republic of Korea; 5 Workplace Mental Health Institute, Kangbuk Samsung Hospital, Seoul, Republic of Korea; 6 Medical Research Institute, Kangbuk Samsung Hospital, Sungkyunkwan University School of Medicine, Seoul, Republic of Korea; 7 Department of Social Welfare, Seoul Women's University, Seoul, Republic of Korea; 8 Department of Nuclear Medicine, Kangbuk Samsung Hospital, Sungkyunkwan University School of Medicine, Seoul, Republic of Korea

**Keywords:** Bidirectional association, blood pressure, cohort study, depression, depressive symptoms, hypertension, hypotension

## Abstract

**Aims:**

To evaluate the bidirectional relationship between blood pressure (BP) and depressive symptoms using a large prospective cohort study.

**Methods:**

Prospective cohort study was performed in 276 244 adults who participated in a regular health check-up and were followed annually or biennially for up to 5.9 years. BP levels were categorised according to the 2017 American College of Cardiology and American Heart Association hypertension guidelines. Depressive symptoms were assessed using Centre for Epidemiologic Studies-Depression (CESD) questionnaire and a cut-off score of ≥25 was regarded as case-level depressive symptoms.

**Results:**

During 672 603.3 person-years of follow-up, 5222 participants developed case-level depressive symptoms. The multivariable-adjusted hazard ratios (HRs) [95% confidence interval (CI)] for incident case-level depressive symptoms comparing hypotension, elevated BP, hypertension stage 1 and hypertension stage 2 to normal BP were 1.07 (0.99–1.16), 0.93 (0.82–1.05), 0.89 (0.81–0.97) and 0.81 (0.62–1.06), respectively (*p* for trend <0.001). During 583 615.3 person-years of follow-up, 27 787 participants developed hypertension. The multivariable-adjusted HRs (95% CI) for incident hypertension comparing CESD 16–24 and ⩾25 to CESD < 16 were 1.05 (1.01–1.11) and 1.12 (1.03–1.20), respectively (*p* for trend <0.001) and in the time-dependent models, corresponding HRs (95% CI) were 1.12 (1.02–1.24) and 1.29 (1.10–1.50), respectively (*p* for trend <0.001).

**Conclusions:**

In this large cohort study of young and middle-aged individuals, higher BP levels were independently associated with a decreased risk for developing case-level depressive symptoms and depressive symptoms were also associated with incident hypertension. Further studies are required to elucidate the mechanisms underlying the bidirectional association between BP levels and incident depression.

## Introduction

Hypertension is a major contributing factor to cardiovascular disease (CVD) and a leading cause of morbidity and mortality worldwide (Lewington *et al*., 2002). Depression is also common and is projected to be the second leading cause of disability-adjusted life-year loss by 2020 (World Health Organization, 2017). In Korea, nationwide epidemiologic surveys have reported a gradual increase in the lifetime prevalence of major depressive disorder over time (2001, 4.0%; 2006, 5.6%; 2011, 6.7%) (Cho *et al*., 2010, 2015). Both diseases are commonly found to coexist, and treatment for hypertension has been reported to affect depression and vice versa.

Previous epidemiological studies have evaluated the temporal association between blood pressure (BP), hypertension and depression, yielding inconsistent results. Some cohort studies have reported no association of high BP with incident depression (Akbaraly *et al*., 2009; Takeuchi *et al*., 2009), whereas others have reported that baseline low BP was associated with a higher risk of developing depression (Paterniti *et al*., 2000; Pulkki-Raback *et al*., 2009; Hiles *et al*., 2016). In a reverse directional relationship, some studies found that depressive symptoms or clinical diagnosis of depression at baseline were associated with an increased risk of hypertension (Jonas *et al*., 1997; Davidson *et al*., 2000; Meyer *et al*., 2004; Nabi *et al*., 2011), while others found that depressive symptoms were associated with a decrease in BP levels over time (Hildrum *et al*., 2008, 2011). These previous studies differed in many aspects, for example, with regard to the study population, its age and sex composition, the period of follow-up, and covariates were considered. Most previous studies were limited by small sample size and lacked consideration of the full spectrum of BP levels, used self-reported hypertension, or did not focus on BP or hypertension as either the main exposure or main outcome (Meyer *et al*., 2004; Akbaraly *et al*., 2009; Pulkki-Raback *et al*., 2009; Takeuchi *et al*., 2009; Hiles *et al*., 2016). Another reason for the discrepancy in previous results lies in the measures of depression (Jorm, 2000; Toker *et al*., 2008) such as clinical diagnosis of depression using interview (Meyer *et al*., 2004; Takeuchi *et al*., 2009; Hiles *et al*., 2016) or depressive symptoms using depression scale (Jonas *et al*., 1997; Davidson *et al*., 2000; Hildrum *et al*., 2008, 2011; Akbaraly *et al*., 2009; Pulkki-Raback *et al*., 2009; Nabi *et al*., 2011).

Coexistence of both depression and hypertension could be explained by depression as a consequence of hypertension or as a risk factor for developing hypertension, or the two conditions may have common pathophysiology and manifest together; however, their temporal and causal relationship remains still unclear. Additionally, the association between BP and depression could be confounded by various factors such as the presence of risky behaviours (e.g., smoking status, alcohol intake), physical activity, body mass index (BMI) and other comorbid conditions (e.g., dyslipidemia, diabetes, insulin resistance and inflammation) (Song *et al*., 2018). These factors should be taken into account to examine an independent association between BP and depression.

Until now, the temporal relationship of BP and depression to each other remains controversial, and, to the best of our knowledge, no longitudinal cohort studies have evaluated a bidirectional association between BP level and depression within the same cohort. Accordingly, we examined the bidirectional relationship between BP and depressive symptoms in a large prospective cohort of young and middle-aged adults while accounting for a wide range of confounding factors.

## Methods

### Setting and study population

This study was part of the Kangbuk Samsung Health Study, a cohort study of South Korean adults who attended a health check-up program annually or biennially at Kangbuk Samsung Hospital Total Healthcare Screening Centres in Seoul and Suwon, South Korea (Chang *et al*., 2016). Over 80% of participants were employees of various companies and local governmental organisations and their spouses. All employees were required to take part in free annual or biennial health examinations according to the Industrial Safety and Health Law in South Korea. The remaining participants voluntarily underwent health examination at one of the two healthcare centres.

**Fig. 1.**
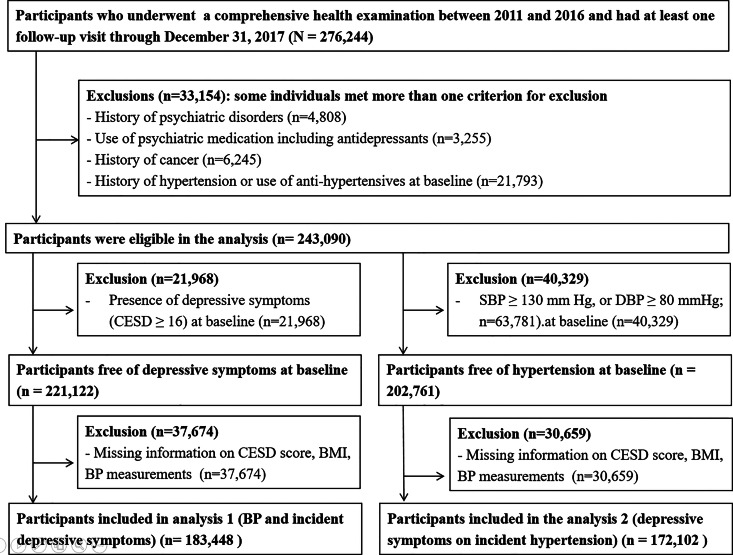
Flow diagram of study participants. CESD, Centre for Epidemiologic Studies-Depression scale; BMI, body mass index; SBP, systolic blood pressure; DBP, diastolic blood pressure.

The present analysis included participants who received a comprehensive health examination between January 2011 and December 2016 (*N* = 417 878). Among them, 276 244 (66.1%) had at least one follow-up visit up to 2017 and were eligible for this cohort study (Fig. 1). We excluded 33 154 participants due to the following at baseline: a history of psychiatric disorders (panic disorder and depression, among others, as well as narcolepsy or insomnia); medication with psychiatric drugs (sedatives, anxiolytics, antidepressants, sleeping pills or other psychiatric drugs); a history of cancer; or a history of hypertension or use of anti-hypertensives at baseline. Since some individuals met more than one exclusion criterion, a total of 243 090 participants were eligible for the analysis. Next, in order to examine the possible association of BP level with the development of case-level depressive symptoms, we further excluded participants with depressive symptoms at baseline (defined as a CES-D score ⩾16) or those with missing information on the Centre for Epidemiologic Studies-Depression (CESD) questionnaire, body mass index (BMI) or BP, leaving 183 448 in the analysis of an association between BP levels and incident case-level depressive symptoms.

For the analysis of the impact of depressive symptoms on incident hypertension, out of 243 090 participants, we further excluded participants with BP ⩾130/80 mm Hg at baseline or missing information, leaving 172 102 participants.

The study was approved by the Institutional Review Board of Kangbuk Samsung Hospital (IRB no. KBSMC 2013-01-217), which waived the informed consent requirement as we only used de-identified data routinely collected in health screening check-ups.

### Measurements

Information regarding sociodemographic characteristics, health behaviours, dietary intake, and medical history was collected through standardised self-administered questionnaires. Physical activity levels were assessed using the validated Korean version of the International Physical Activity Questionnaire (IPAQ) Short Form and categorised into inactive, minimally active, or health-enhancing physically active (HEPA) (Craig *et al*., 2003; Chun, 2012). History of CVD was defined as participants who reported physician-diagnosed CVD including angina/myocardial infarction and stroke (ischemic or haemorrhagic).

Anthropometric parameters were measured by trained nurses. Blood pressure was measured using an automated oscillometric device (53000; Welch Allyn, New York, NY, USA) by trained nurses while participants were in a sitting position with their arms supported at heart level. Three consecutive BP readings were obtained after the participants had been resting quietly in a sitting position for 5 min. The average of the second and third BP readings was used in the analysis. For the model for incident depressive symptoms, BP was classified according to the 2017 American College of Cardiology (ACC) and American Heart Association (AHA) hypertension guidelines (Reboussin *et al*., 2018) as follows: normal BP (SBP < 120 and DBP < 80 mm Hg), elevated BP (SBP 120–129 and DBP < 80 mm Hg), hypertension stage 1 (SBP 130–139 or DBP 80–89 mm Hg), and hypertension stage 2 (SBP ⩾ 140 or DBP ⩾ 90 mm Hg). Since previous studies reported an effect of hypotension on depression (Paterniti *et al*., 2000; Herva *et al*., 2006; Hiles *et al*., 2016), we further included a low BP category (SBP < 90 or DBP < 60 mm Hg). For the incident hypertension model, hypertension was defined as current use of antihypertensive medications, SBP ⩾ 130 mm Hg, or DBP ⩾ 80 mm Hg.

Fasting blood measurements included glucose, insulin, lipid profile, and highly sensitive C-reactive protein (hsCRP). The homeostasis model assessment of insulin resistance (HOMA-IR) was calculated as (fasting glucose × fasting insulin)/22.5. Diabetes was defined as fasting serum glucose ⩾126 mg/dl, A_1_c level ⩾6.5%, self-reported use of insulin, or antidiabetic medications.

Depressive symptoms were assessed at baseline and subsequent follow-up visits using the validated Korean version of the CESD scale (Cho and Kim, 1998): the internal consistency of the Korean version was 0.89 and two optimal cut-off points were suggested including 24/25, the point which best corresponded to the clinical diagnosis of depression, and 20/21, which most effectively detected depressive symptoms during screening (Cho and Kim, 1998). The higher cut-off points in Korea than in Western countries are possibly due to the influence of Confucianism and its values of modesty, where a reluctance to express positive emotions (such as happiness and satisfaction) can be indicative of cultural norms rather than depressive symptoms (Cho and Kim, 1998). On the other hand, a CESD score of 16 has been traditionally used as an optimal CESD cutoff for the detection of depressive symptoms (Roberts *et al*., 1990). Therefore, the cutoff values used were a CESD score <16 to rule out depressive symptoms at baseline and a CESD score ≥25 to rule in case-level depressive symptoms at follow-up visits.

### Statistical analysis

Descriptive statistics were used to summarise baseline characteristics of study participants according to the BP category. To test for linear trends, the median value of each BP category was treated as a continuous variable in the models.

First, we evaluated associations between baseline BP levels and incident case-level depressive symptoms during follow-up. The study endpoint was the new onset of case-level depressive symptoms. Participants were followed from the baseline visit to the visit of onset of case-level depressive symptoms or to the final examination conducted prior to December 31, 2017. Since the onset of case-level depressive symptoms occurred at an unknown time point between the visit when depression was identified and the previous visit, we used parametric proportional hazards models to account for interval-censored events (Royston and Parmar, 2002; Lambert and Royston, 2009). In these models, the baseline hazard function was parameterised with restricted cubic splines in log time with four degrees of freedom. Incidence density was calculated as the number of incident cases divided by person-years during the follow-up period. We calculated hazard ratios (HRs) and 95% confidence intervals (CIs) for incident case-level depressive symptoms according to BP categories. The reference group was defined as participants with normal BP (SBP 90–119 & DBP 60–79 mm Hg). The proportional hazards assumption was assessed by examining the estimated log (-log) survival graphs; no violation of the assumption was found. To further explore the shape of the dose-response relationship of SBP or DBP levels with the development of case-level depressive symptoms, restricted cubic splines with knots were used at the 5th, 27.5th, 50th, 72.5th and 95th percentiles of SBP or DBP level distributions.

We also evaluated associations of depressive symptoms at baseline with incident hypertension during follow-up. For this analysis, we categorised participant CESD score into three groups based on the cut-off scores (Cho and Kim, 1998): CESD score <16 (no depressive symptoms); CESD 16–24 (mild to moderate depressive symptoms); and CESD ⩾ 25 (severe depressive symptoms). A cut-off score of ⩾25 was regarded as case-level depressive symptoms.

To control for potential confounders, models were initially adjusted for age and sex and then further adjusted for health centre (Seoul or Suwon), year of screening exam, BMI, smoking status (never, past, current, or unknown), alcohol intake (0, <20, ⩾20 g/day, or unknown), physical activity (inactive, minimally active, HEPA, or unknown), educational level (high school graduate or less, community college or university graduate, graduate school or higher, or unknown), total calorie intake (in quintile or missing) and history of diabetes. Moreover, to evaluate the effects of changes in BP levels, CESD score, and other covariates over follow-up, we conducted additional analyses, introducing the main exposure of interest (BP or CESD categories) and other covariates as a time-varying covariate in the models.

All *p* values were two-tailed, and values of *p* < 0.05 were considered statistically significant. We used STATA version 15.0 (Stata Corp., College Station, TX, USA) for data analysis.

## Results

The mean (s.d.) baseline age of 183 448 participants without baseline depressive symptoms was 38.2 years (7.3) and 57% were male (Table 1). BP categories were positively associated with age, male sex, current smoker, alcohol intake, obesity, diabetes, BMI, glucose, triglyceride, LDL-C, total cholesterol, hsCRP and HOMA-IR and negatively associated with high education level and HDL-C.

**Table 1.** Baseline characteristics according BP categories

Characteristics	Overall	BP categories					*p* for trend
		SBP < 90 or DBP < 60	SBP 90–119 & DBP 60–79	SBP 120–129 & DBP 60–79	SBP 130–139 or DBP 80–89	SBP ⩾ 140 or DBP ⩾ 90	
Naming of BP categories		Hypotension	Normal BP	Elevated BP	Hypertension stage 1	Hypertension stage 2	
Number	183 448	24 737	116 754	11 229	27 931	2797	
Age, years^a^	38.2 (7.3)	35.8 (6.4)	38.0 (7.1)	38.3 (8.0)	40.5 (7.4)	42.2 (7.3)	<0.001
Male (%)	57.0	16.7	55.8	83.3	84.3	84.5	<0.001
Current smoker (%)	27.7	9.6	27.3	37.3	40.1	39.6	<0.001
Alcohol intake (%)^b^	22.3	7.6	20.1	32.1	37.6	43.6	<0.001
HEPA (%)^c^	15.3	12.9	15.1	20.8	16.2	16.2	<0.001
High education level (%)^d^	85.6	86.1	85.9	85.5	84.3	82.8	<0.001
Diabetes (%)	2.6	0.6	2.1	3.7	5.2	8.2	<0.001
Obesity (%)^e^	26.3	7.6	22.8	46.3	46.2	55.9	<0.001
Body mass index (kg/m^2^)	23.1 (3.2)	21.2 (2.6)	22.9 (3.0)	25.0 (3.2)	24.9 (3.2)	25.8 (3.5)	<0.001
Glucose (mg/dl)^a^	94.4 (12.8)	89.1 (8.5)	93.7 (11.6)	97.5 (13.4)	99.9 (16.2)	103.7 (22.9)	<0.001
Total cholesterol (mg/dl)^a^	193.8 (33.9)	181.8 (31.1)	192.5 (32.9)	201.1 (34.8)	205.5 (34.8)	210.9 (37.6)	<0.001
LDL-C (mg/dl)^a^	120.1 (31.7)	106.4 (28.0)	119.1 (30.9)	128.7 (32.0)	131.3 (32.2)	135.5 (33.2)	<0.001
HDL-C (mg/dl)^a^	58.6 (15.0)	64.6 (14.6)	59.0 (15.0)	54.6 (13.8)	54.0 (14.0)	53.2 (13.8)	<0.001
Triglycerides (mg/dl)	89 (64–133)	65 (51–88)	87 (63–126)	109 (78–160)	124 (87–179)	136 (95–196)	<0.001
hsCRP (mg/l)^f^	0.4 (0.2–0.9)	0.3 (0.2–0.6)	0.4 (0.2–0.8)	0.5 (0.3–1.1)	0.6 (0.3–1.1)	0.7 (0.4–1.4)	<0.001
HOMA-IR^f^	1.17 (0.77–1.73)	0.93 (0.62–1.34)	1.14 (0.76–1.66)	1.38 (0.91–2.04)	1.48 (0.98–2.20)	1.75 (1.16–2.63)	<0.001
Total energy intake (kcal/d)^f^^,^^g^	1546.0 (1196.9–1928.4)	1434.3 (1081.6–1809.3)	1543.3 (1193.6–1923.4)	1654.8 (1311.9–2057.8)	1609.2 (1286.8–1991.3)	1588.3 (1248.8–1957.5)	<0.001

BP, blood pressure; DBP, diastolic blood pressure; HDL-C, high-density lipoprotein-cholesterol; HEPA, health-enhancing physical activity; hsCRP, high sensitivity C-reactive protein; HOMA-IR, homeostasis model assessment of insulin resistance; LDL-C, low-density lipoprotein cholesterol; SBP, systolic blood pressure.

Data are expressed as

a mean (standard deviation).

b ⩾ 20 g of ethanol per day.

c defined as physical activity that meets either of two criteria: (i) vigorous intensity activity on 3 or more days per week accumulating ⩾1500 metabolic equivalent (MET) min/week; or (ii) 7 days of any combination of walking, moderate intensity, or vigorous intensity activities achieving at least 3000 MET min/week.

d ⩾ College graduate.

e BMI ⩾25 kg/m^2^.

f median (interquartile range), or percentage.

g Among 129 927 participants with plausible estimated energy intake levels (within three standard deviations from the log-transformed mean energy intake).

**Fig. 2.**
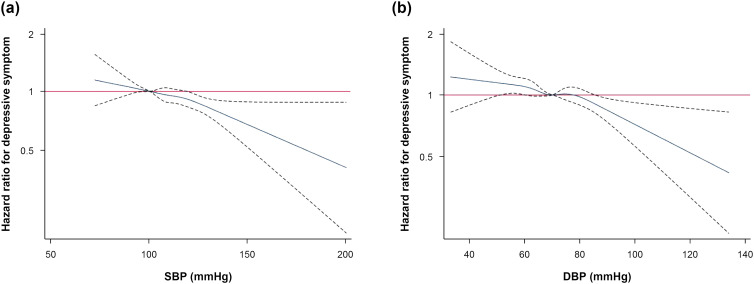
Hazard ratios for incident case-level depressive symptoms by levels of systolic blood pressure (*a*) and diastolic blood pressure (*b*). Curves represent adjusted hazard ratios (blue lines) and their 95% CI (dashed lines). The reference values (red lines) were set as participants with systolic blood pressure 90–119 (*a*) or diastolic blood pressure 60–79 (*b*). Model was adjusted for age, sex, centre, year of screening exam, BMI, smoking status, alcohol intake, physical activity, educational level, total calorie intake and history of diabetes. SBP, systolic blood pressure; DBP, diastolic blood pressure.

During 672 603.3 person-years with a median follow-up of 3.9 years (interquartile range, 2.1–5.0; maximum 5.9 years), 5222 participants developed case-level depressive symptoms (incidence rate 7.8 per 1000 person-years; Table 2). After adjustment for confounders, multivariable-adjusted HRs (95% CIs) for incident case-level depressive symptoms comparing hypotension, elevated BP, hypertension stage 1 and hypertension stage 2 to normal BP were 1.07 (0.99–1.16), 0.93 (0.82–1.05), 0.89 (0.81–0.97) and 0.81 (0.62–1.06), respectively (*p* for trend <0.001). In multivariable-adjusted spline regression models, both SBP and DBP were inversely associated with incident case-level depressive symptoms (Fig. 2). When BP categories and covariates during follow-up were updated as time-varying covariates, the inverse association between BP categories and incident case-level depressive symptoms persisted. In sensitivity analyses using the criterion of depressive symptoms of either CESD ⩾ 16 or CESD ⩾ 25, the inverse association between BP category and incident depressive symptoms was consistently observed (online Supplementary Tables 1, 2). Associations between BP category and incident case-level depressive symptoms did not significantly differ across clinically various subgroups and age strata of study participants (online Supplementary Tables 3, 4).

**Table 2.** Development of case-level depressive symptoms by BP categories among 183 448 subjects free of depressive symptoms at baseline

BP categories	Naming of BP categories	Person-years	Incident cases	Incidence density (per 1000 person-years)	Age-sex adjusted HR (95% CI)	Multivariable-adjusted HR (95% CI)^a^ Model 1	HR (95% CI)^b^ in model using time-dependent variables
SBP < 90 or DBP < 60	Hypotension	83 472.6	832	10.0	1.07 (0.99–1.16)	1.07 (0.99–1.16)	1.05 (0.97–1.13)
SBP 90–119 & DBP 60–79	Normal BP	426 037.6	3383	7.9	1.00 (reference)	1.00 (reference)	1.00 (reference)
SBP 120–129 & DBP 60–79	Elevated BP	43 192.8	285	6.6	0.93 (0.83–1.06)	0.93 (0.82–1.05)	1.08 (0.95–1.24)
SBP 130–139 or DBP 80–89	Hypertension stage 1	109 633.8	669	6.1	0.89 (0.82–0.97)*	0.89 (0.81–0.97)*	0.94 (0.86–1.03)
SBP ⩾ 140 or DBP ⩾ 90	Hypertension stage 2	10 266.5	53	5.2	0.81 (0.62–1.07)	0.81 (0.62–1.06)	0.77 (0.60–0.99)*
*p* for trend					<0.001	<0.001	0.028

BP, blood pressure; CESD, Centre for Epidemiologic Studies-Depression scale; CI, confidence intervals; DBP, diastolic blood pressure; HR, hazard ratio; SBP, systolic blood pressure.

a Estimated from parametric proportional hazard model. Multivariable model was adjusted for age, sex, centre, year of screening exam, BMI, smoking status, alcohol intake, physical activity, educational level, total calorie intake, history of diabetes and baseline CESD.

b Estimated from parametric proportional hazard models with BP categories, smoking status, alcohol intake, physical activity, BMI, total calorie intake and baseline CESD as time-dependent variables and baseline age, sex, centre, year of screening exam, education level and history of diabetes as time-fixed variables.

*Statistically significant differences (*p* < 0.05).

The baseline prevalence of CESD score <16, CESD 16–24 and CESD ⩾ 25 was 88.7%, 7.8% and 3.4%, respectively (online Supplementary Table 5). During 583 615.3 person-years with a median follow-up of 3.6 years (interquartile range, 1.9–5.0 years), 27 787 participants developed new-onset hypertension (rate 47.6 per 1000 person-years; Table 3). The multivariable-adjusted HRs (95% CI) comparing CESD 16–24 and CESD ⩾25 to CESD <16 were 1.05 (1.01–1.11) and 1.12 (1.03–1.20), respectively. In the time-dependent models, corresponding HRs (95% CI) were 1.12 (1.02–1.24) and 1.29 (1.10–1.50), respectively (*p* for trend <0.001). When CESD score was modelled continuously, the HRs for hypertension per 5-point increase in CESD score were 1.01 (1.003–1.02) in a multivariable-adjusted model and 1.06 (1.04–1.08) in a time-dependent model. In analyses after excluding participants newly taking either anti-depressant or anti-hypertensives during follow up, similar findings were observed (online Supplementary Tables 6, 7).

**Table 3.** Development of hypertension by CESD categories among 172 102 subjects free of hypertension at baseline

CESD categories	Naming of CESD categories	Person-years	Incident cases	Incidence density (per 1000 person-years)	Age-sex adjusted HR (95% CI)	Multivariable-adjusted HR (95% CI)^a^ Model 1	HR (95% CI)^b^ in model using time-dependent variables
<16	No depressive symptoms	517 819.6	25 312	48.9	1.00 (reference)	1.00 (reference)	1.00 (reference)
16–24	Mild to moderate depressive symptoms	45 549.2	1779	39.1	1.05 (1.002–1.10)*	1.05 (1.01–1.11)*	1.12 (1.02–1.24)*
⩾25	Severe depressive symptoms	20 246.5	696	34.4	1.09 (1.01–1.18)*	1.12 (1.03–1.20)*	1.29 (1.10–1.50)*
*p* for trend					0.003	<0.001	<0.001
Per 5 score increase in CESD					1.01 (1.003–1.02)*	1.01 (1.003–1.02)*	1.06 (1.04–1.08)*

CESD, Centre for Epidemiologic Studies-Depression scale; CI, confidence intervals; HR, hazard ratio.

a Estimated from parametric proportional hazard model. Multivariable model 1 was adjusted for age, sex, centre, year of screening exam, BMI, smoking status, alcohol intake, physical activity, educational level, total calorie intake, history of diabetes and baseline SBP.

b Estimated from parametric proportional hazard models with CESD categories, smoking status, alcohol intake, physical activity, BMI, total calorie intake and baseline SBP as time-dependent variables and baseline age, sex, centre, year of screening exam, education level and history of diabetes as time-fixed variables.

*Statistically significant differences (*p* < 0.05).

To account for potential differences between participants with follow-up visits and those with no follow-up visit (online Supplementary Table 8), the results using inverse probability weight analysis were similar to the original results (online Supplementary Tables 9, 10). Additionally, to account for the missing values (online Supplementary Tables 11, 12), we also performed missing value analysis with multiple imputations (*m* = 20 imputations) and found similar results (online Supplementary Tables 13, 14).

## Discussion

In this large prospective cohort study of young and middle-aged individuals, higher BP at baseline was associated with a decreased risk of incident case-level depressive symptoms. This association remained significant after adjusting for possible confounders. In the other direction, depressive symptoms at baseline were associated with an increased risk of hypertension, indicating that BP and depressive symptoms may affect each other.

Previous studies have evaluated an association between BP and depressive symptoms or clinical diagnosis of depression, reporting conflicting results. A cohort study by Akbaraly *et al*. (2009) of 5232 middle-aged participants from the Whitehall II prospective cohort study found no association between high BP and new-onset depressive symptoms 6 years later. Another cohort study by Takeuchi *et al*. (2009) of 956 young and middle-aged Japanese male employees similarly demonstrated no association between high BP (as one component of metabolic syndrome) and incident diagnosed depression in consequent years. In these two studies, high BP was defined as SBP ⩾ 130 or DBP ⩾ 85 mm Hg without consideration of the full range of BP.

On the other hand, our findings are consistent with some prospective cohort studies. A population-based prospective cohort study of 921 participants by Pulkki-Raback *et al*. (2009) examined the bidirectional association between depressive symptoms and metabolic syndrome in childhood and adulthood and found that higher BP as a continuous variable in childhood predicted lower future depressive symptoms in men with a mean age of 33 years. Hiles *et al*. (2016) also reported a consistent, non-significant inverse association of SBP as a continuous variable with newly diagnosed depression across 2- and 6-year follow-up waves in adults under 65. In all previous studies, the primary exposure of interest was metabolic syndrome; thus, a detailed analysis of the full range of BP and associations with incident depression was not performed since high BP was just one metabolic syndrome component.

In our study, hypotension tended to be weakly associated with later onset of case-level depressive symptoms compared with normal BP, but this association did not reach statistical significance. Similarly, Paterniti *et al*. (2000) reported a temporal relationship between low BP and incident depressive symptoms after 2-year follow-up in the elderly and showed that low baseline DBP and a decrease in BP over time were predictors of new-onset depressive symptoms. Another longitudinal cohort study found that lower systolic BP predicts new-onset diagnosed depression in the offspring of parents with depression (Hammerton *et al*., 2013).

In a meta-analysis of nine prospective studies (Meng *et al*., 2012), positive associations between depression and incident hypertension were observed. Hypertension is a relatively stable and chronic disease over time, whereas depression is an episodic disorder that repeats in development, improvement and recurrence (Solomon *et al*., 2000; Nabi *et al*., 2011) with more dynamic changes in symptoms over time. In our analysis using a time-dependent model with consideration of changing depressive symptoms over time, case-level depressive symptoms were consistently associated with an increased risk of incident hypertension.

The mechanisms underlying the relationship between higher BP and lower risk of depression are not yet fully understood. BP and depression could be causally related, or the association of both conditions could be due to shared common risk factors or secondary to a third factor. Activity of the central monoamine system (Stahl, 2000) or neuropeptide Y (Michalkiewicz *et al*., 2003; Karl and Herzog, 2007) offers possible sources of a common factor. Based on the considerable interconnections between locus coeruleus - norepinephrine (NE) neurons and raphe – serotonin (5-HT) neurons in the brain (Ressler and Nemeroff, 2000), boosting of one or both monoamines by SSRI or SNRI antidepressants may induce high BP and clinical improvement of depression (Ressler and Nemeroff, 1999; Watts *et al*., 2012). When depletion of these monoamines is induced, common symptoms of low BP and the development of depression are observed (Ressler and Nemeroff, 1999; Watts *et al*., 2012). The autonomic neural control of cardiovascular function involves a number of neuropeptides. Abnormal alterations of neuropeptide Y may explain the association between abnormal BP and depression. Neuropeptide Y, an important neurotransmitter of NE signalling, seems to lower sympathetic outflow and decrease BP (McDermott *et al*., 1993). Meanwhile, depression is characterised by altered levels of neuropeptide Y (Hashimoto *et al*., 1996). It has been suggested that impaired metabolism of plasma neuropeptide Y in depressed patients could be involved in the pathogenesis of depression (Heilig, 2004).

A possible explanation of the relationship between lower BP and increasing risk of depression is that chronic symptoms observed in hypotension such as weakness, fatigue and dizziness could lead to psychological stress and subsequent depression (Wessely *et al*., 1990; Pilgrim *et al*., 1992). In addition, the genetic correlation of depression with hypertension is estimated to be 19% (Scherrer *et al*., 2003), suggesting the influence of pleiotropic genes and shared biological pathways among them. Future studies that assess these factors will be required to elucidate the mechanism underlying the inverse association between BP and incident depression.

The association between physical health and depression can differ by age groups (Fiske *et al*., 2009; Nabi *et al*., 2011). In our study, the relationship between BP and depressive symptoms did not significantly differ by age groups. Even though our study population is large, the number of participants of each exposure category among extreme age groups (i.e., aged <30 years and aged ⩾50 years) and incident cases is relatively small, resulting in unreliable estimates. Further research is required to investigate the age difference on the association between depression and BP with a large, population-based longitudinal sample covering a wide range of ages.

There are also several plausible mechanisms through which depressive symptoms could increase the risk of subsequent hypertension. First, the dysregulation of adrenergic activities is often observed in those with clinical depression (Siever and Davis, 1985). Decreased heart rate variability reflecting alterations in para- and sympathetic tone have been also found in depressed individuals (Light *et al*., 1983; Siever and Davis, 1985). Such adrenergic alternation may play a role in sustaining BP elevations over time. Second, the association of depression with various health behaviours could then impact the risk of hypertension (Jones-Webb *et al*., 1996; Son *et al*., 1997). However, adjustment for BMI, smoking status, alcohol intake, physical activity, and total calorie intake did not alter the results in our study.

There are several limitations to our study. First, depressive symptoms were only assessed using CESD score, which is not the gold standard for the clinical diagnosis of depression. However, CESD questionnaires are well established for use in population studies and have a good correlation with clinical diagnoses of depression (Dayan and Panicker, 2013). Second, the determination of BP was based on a single-day measurement, although three readings were taken. If the degree of misclassification did not differ in relation to depressive symptoms, this type of error was likely non-differential, resulting in an underestimation of the association between BP and depression. Third, due to lack of information on the specific medication class, its dosage and duration of use in our dataset, the effect of specific antidepressant on BP and vice versa could not be examined. Additionally, despite adjustment for multiple covariates in our analyses, we cannot rule out the possibility of some unmeasured or residual confounding factors associated with both BP and depression. Lastly, the study participants were mainly young and middle-aged, relatively healthy Korean adults, which may limit the generalisability of our findings to other populations with different characteristics such as older age groups and other race/ethnicity groups. Despite these limitations, our study has notable strengths, including a prospective cohort study with a bidirectional approach, a large sample size, availability of detailed information on clinical and laboratory data and repeated measures at baseline and follow-up visits, enabling us to examine the temporal and independent relationship between BP and depression while considering the full spectrum of BP levels, along with time-dependent measures where updated status of BP and depressive symptoms during follow-up could be incorporated in a time-dependent model.

The significance of our findings lies in intriguing research on the underlying biological mechanisms for the inverse association between BP levels and incident depression. Several anti-hypertensives have been reported as potential risk factors for depression in multiple studies over the past decades (Patten, 1990; Hallas, 1996; Rathmann *et al*., 1999). It has been disputed whether this is due to antihypertensive treatment, a close link between hypertension itself and depression, or both. The present study findings raise the question of whether the occurrence of depression during treatment of hypertension may be attributable to the inherent connection between BP levels and depression. Our findings support the determination of medication choice in patients on antihypertensive treatment based on cardiovascular risk, but not postponement or discontinuation for possible side effects of depression. Targeting blood-vessel, specific drugs might be helpful for controlling BP without inducing depression in the future as well.

In conclusion, in this large cohort study, increased BP levels were independently associated with a decreased risk of case-level depressive symptoms, while depressive symptoms were significantly associated with incident hypertension. A better understanding of the interrelationship between BP and depression may help identify and manage people at high risk for depression or hypertension.

## Supplementary material

For supplementary material accompanying this paper visit http://dx.doi.org/10.1017/S2045796020000542. click here to view supplementary material

## Data

The data are not available to be shared publicly because we do not have permission from the IRB to distribute the data. However, analytical methods are available from the corresponding author on reasonable request.
