# Supplementary material for: Bidirectional association between blood pressure and depressive symptoms in young and middle-age adults: A cohort study
Source: Epidemiol Psychiatr Sci. 2020 Jul 15;29:e142. doi: 10.1017/S2045796020000542 (PMC7372173; doi:10.1017/S2045796020000542)
Supplement: Supplementary file 1 [file S2045796020000542sup001.docx]

**Supplemental Materials**

**Bidirectional association between blood pressure and depressive symptoms in young and middle age adults: A cohort study**

**Online Tables**

**Supplementary Table 1.** Development of depressive symptoms (CESD score of 16 or greater) by BP categories among 183,448 subjects free of depressive symptoms (CESD score of 16 or greater) at baseline

**Supplementary Table 2.** Development of case-level depressive symptoms (CESD score of 25 or greater) by BP categories among 198,783 subjects free of case-level depressive symptoms (CESD score of 25 or greater) at baseline

**Supplementary Table 3.** Hazard ratios (95% CI) of incident case-level depressive symptoms according to BP categories in clinically relevant subgroups

**Supplementary Table 4.** Hazard ratios (95% CI) of incident case-level depressive symptoms according to BP category in age strata

**Supplementary Table 5.** Baseline characteristics according to CESD categories

**Supplementary Table 6.** Development of case-level depressive symptoms by BP categories after excluding 807 subjects taking new anti-depressant use or 4,186 subjects taking new anti-hypertensive medication use during follow up

**Supplementary Table 7.** Development of hypertension by CESD categories after excluding 955 subjects taking new anti-depressant use or 1,116 subjects taking new anti-hypertensive medication use during follow up

**Supplementary Table 8.** Baseline characteristics according to follow up loss

**Supplementary Table 9.** Development of case-level depressive symptoms by BP categories using inverse probability weights.

**Supplementary Table 10.** Development of hypertension by CESD categories using inverse probability weights.

**Supplementary Table 11.** Baseline characteristics according to exclusion in the model for development of case-level depressive symptoms by BP category

**Supplementary Table 12.** Baseline characteristics according to exclusion in the model for incident hypertension by CESD category

**Supplementary Table 13.** Development of case-level depressive symptoms by BP category using missing value analysis.

**Supplementary Table 14.** Development of hypertension by CESD category using missing value analysis.

**Supplementary Table 1.** Development of depressive symptoms (CESD score of 16 or greater) by BP categories among 183,448 subjects free of depressive symptoms (CESD score of 16 or greater) at baseline

| BP categories |  | Multivariable-adjusted HR (95% CI)^a^ | |
| --- | --- | --- | --- |
| SBP <90 or DBP <60 | Hypotension | 1.06 (1.02-1.10) | |
| SBP 90-119 & DBP 60-79 | Normal BP | 1.00 (reference) | |
| SBP 120-129 & DBP 60-79 | Elevated BP | 0.96 (0.90-1.015) | |
| SBP 130-139 or DBP 80-89 | Hypertension stage 1 | 0.91 (0.87-0.95) | |
| SBP ≥140 or DBP ≥90 | Hypertension stage 2 | 0.87 (0.76-0.98) | |
| *p* for trend |  | | <0.001 |

Abbreviations: BP, blood pressure; CESD, Center for Epidemiologic Studies-Depression scale; CI, confidence intervals; DBP, diastolic blood pressure; HR, hazard ratio; SBP, systolic blood pressure.

^a^ Estimated from parametric proportional hazard model. Multivariable model was adjusted for age, sex, center, year of screening exam, BMI, smoking status, alcohol intake, physical activity, educational level, total calorie intake, history of diabetes and baseline CESD

**Supplementary Table 2.** Development of case-level depressive symptoms (CESD score of 25 or greater) by BP categories among 198,783 subjects free of case-level depressive symptoms (CESD score of 25 or greater) at baseline

| BP categories |  | Multivariable-adjusted HR (95% CI)^a^ | |
| --- | --- | --- | --- |
| SBP <90 or DBP <60 | Hypotension | 1.06 (0.99-1.13) | |
| SBP 90-119 & DBP 60-79 | Normal BP | 1.00 (reference) | |
| SBP 120-129 & DBP 60-79 | Elevated BP | 0.90 (0.81-1.00) | |
| SBP 130-139 or DBP 80-89 | Hypertension stage 1 | 0.91 (0.85-0.98) | |
| SBP ≥140 or DBP ≥90 | Hypertension stage 2 | 0.84 (0.67-1.05) | |
| *p* for trend |  | | <0.001 |

Abbreviations: BP, blood pressure; CESD, Center for Epidemiologic Studies-Depression scale; CI, confidence intervals; DBP, diastolic blood pressure; HR, hazard ratio; SBP, systolic blood pressure.

^a^ Estimated from parametric proportional hazard model. Multivariable model was adjusted for age, sex, center, year of screening exam, BMI, smoking status, alcohol intake, physical activity, educational level, total calorie intake, history of diabetes and baseline CESD

| \| **Supplementary Table 3.** Hazard ratios^a^ (95% CI) of incident case-level depressive symptoms according to BP categories in clinically relevant subgroups \| \| \| \| \| \| \| \| \| \| \| \| \| \| \| --- \| --- \| --- \| --- \| --- \| --- \| --- \| --- \| --- \| --- \| --- \| --- \| --- \| --- \| \| Subgroup \| BP categories \| \| \| \| \| \| \| \| \| \| *p* for trend \| *p* for interaction \| \| \| SBP <90  or DBP <60 \| \| SBP 90-119  & DBP 60-79 \| \| SBP 120-129  & DBP 60-79 \| \| SBP 130-139  or DBP 80-89 \| \| SBP ≥140  or DBP ≥90 \| \| \| Age, years \|  \| \|  \| \|  \| \|  \| \|  \| \|  \| 0.280 \| \| \| <40 (*n*=117,150) \| 1.12 (1.03-1.22) \| \| reference \| \| 0.96 (0.82-1.12) \| \| 0.91 (0.81-1.02) \| \| 0.96 (0.66-1.40) \| \| <0.001 \|  \| \| \| ≥40 (*n*=66,298) \| 0.97 (0.82-1.16) \| \| reference \| \| 0.88 (0.72-1.07) \| \| 0.82 (0.72-0.93)* \| \| 0.63 (0.42-0.94)* \| \| 0.003 \|  \| \| \| Sex \|  \| \|  \| \|  \| \|  \| \|  \| \|  \| 0.764 \| \| \| Women (*n*=78,920) \| 1.06 (0.97-1.15) \| \| reference \| \| 0.83 (0.65-1.07) \| \| 0.87 (0.73-1.03) \| \| 0.97 (0.56-1.68) \| \| 0.027 \|  \| \| \| Men (*n*=104,528) \| 1.12 (0.91-1.37) \| \| reference \| \| 0.97 (0.84-1.12) \| \| 0.90 (0.81-0.99)* \| \| 0.77 (0.56-1.06) \| \| 0.004 \|  \| \| \| BMI, kg/m^2^ \|  \| \|  \| \|  \| \|  \| \|  \| \|  \| 0.345 \| \| \| <25 (*n*=135,269) \| 1.07 (0.98-1.16) \| \| reference \| \| 0.99 (0.85-1.16) \| \| 0.86 (0.77-0.96)* \| \| 0.61 (0.38-0.99)* \| \| <0.001 \|  \| \| \| ≥25 (*n*=48,179) \| 1.04 (0.80-1.35) \| \| reference \| \| 0.86 (0.71-1.04) \| \| 0.92 (0.81-1.05) \| \| 0.95 (0.68-1.33) \| \| 0.214 \|  \| \| \| Current smoking \|  \| \|  \| \|  \| \|  \| \|  \| \|  \| 0.821 \| \| \| No (n=120,752) \| 1.08 (0.99-1.18) \| \| reference \| \| 1.00 (0.84-1.17) \| \| 0.89 (0.79-1.01) \| \| 0.87 (0.60-1.26) \| \| 0.013 \|  \| \| \| Yes (*n*=46,250) \| 1.12 (0.87-1.43) \| \| reference \| \| 0.89 (0.72-1.09) \| \| 0.91 (0.79-1.05) \| \| 0.69 (0.42-1.11) \| \| 0.023 \|  \| \| \| Alcohol intake, g/day \|  \| \|  \| \|  \| \|  \| \|  \| \|  \| 0.231 \| \| \| <20 (*n*=135,127) \| 1.09 (1.00-1.19) \| \| reference \| \| 1.02 (0.88-1.18) \| \| 0.89 (0.80-0.99)* \| \| 0.82 (0.57-1.19) \| \| 0.007 \|  \| \| \| ≥20 (*n*=38,661) \| 0.90 (0.68-1.19) \| \| reference \| \| 0.76 (0.60-0.97)* \| \| 0.89 (0.77-1.03) \| \| 0.82 (0.54-1.25) \| \| 0.034 \|  \| \| \| HEPA \|  \| \|  \| \|  \| \|  \| \|  \| \|  \| 0.217 \| \| \| No (*n*=153,787) \| 1.08 (0.99-1.17) \| \| reference \| \| 0.93 (0.81-1.07) \| \| 0.93 (0.84-1.02) \| \| 0.76 (0.56-1.03) \| \| 0.002 \|  \| \| \| Yes (*n*=27,808) \| 1.03 (0.84-1.28) \| \| reference \| \| 0.94 (0.72-1.24) \| \| 0.71 (0.57-0.89)* \| \| 1.05 (0.58-1.91) \| \| 0.076 \|  \| \| \| HOMA-IR \|  \| \|  \| \|  \| \|  \| \|  \| \|  \| 0.464 \| \| \| <2.5 (*n*=165,252) \| 1.07 (0.99-1.16) \| \| reference \| \| 0.95 (0.83-1.08) \| \| 0.89 (0.81-0.98)* \| \| 0.69 (0.49-0.98)* \| \| <0.001 \|  \| \| \| ≥2.5 (*n*=17,347) \| 1.03 (0.68-1.56) \| \| reference \| \| 0.84 (0.60-1.18) \| \| 0.82 (0.66-1.03) \| \| 1.09 (0.69-1.73) \| \| <0.001 \|  \| \| \| hsCRP, mg/L \|  \| \|  \| \|  \| \|  \| \|  \| \|  \| 0.460 \| \| \| <1.0 (*n*=117,994) \| 1.10 (1.01-1.21) \| \| reference \| \| 0.91 (0.78-1.07) \| \| 0.83 (0.74-0.92)* \| \| 0.66 (0.43-1.01) \| \| <0.001 \|  \| \| \| ≥1.0 (*n*=33,604) \| 1.05 (0.85-1.29) \| \| reference \| \| 0.98 (0.77-1.25) \| \| 0.97 (0.83-1.15) \| \| 0.64 (0.37-1.08) \| \| 0.173 \|  \| \| \| History of CVD \|  \| \|  \| \|  \| \|  \| \|  \| \|  \| 0.439 \| \| \| No (n=182,349) \| 1.07 (0.99-1.16) \| \| reference \| \| 0.93 (0.82-1.05) \| \| 0.89 (0.81-0.97)* \| \| 0.80 (0.61-1.05) \| \| <0.001 \|  \| \| \| Yes (*n*=1,099) \| 0.70 (0.16-2.97) \| \| reference \| \| 1.54 (0.53-4.48) \| \| 0.58 (0.20-1.69) \| \| 2.94 (0.39-21.85) \| \| 0.906 \|  \| \| \| Abbreviations: BMI, body mass index; BP, blood pressure; CI, confidence intervals; CVD, cardiovascular disease; DBP, diastolic blood pressure; HEPA, health-enhancing physical activity; HOMA-IR, homeostasis model assessment of insulin resistance; hsCRP, high sensitivity C-reactive protein; SBP, systolic blood pressure. *Statistically significant differences (p<0.05).  History of CVD was defined as participants who reported physician-diagnosed CVD including angina/myocardial infarction and stroke (ischemic or hemorrhagic).  ^a^Estimated from parametric proportional hazard models adjusted for age, center, year of screening exam, smoking status, alcohol intake, total energy intake, physical activity, education level, history of diabetes and baseline CESD. \| \| \| \| \| \| \| \| \| \| \| \| \| \| \| **Supplementary Table 4.** Hazard ratios^a^ (95% CI) of incident case-level depressive symptoms according to BP category in age strata \| \| \| \| \| \| \| \| \| \| \| \| \| \| \| Age, years \| \| BP categories \| \| \| \| \| \| \| \| \| \| \| p for trend \| \| SBP <90  or DBP <60 \| \| SBP 90-119  & DBP 60-79 \| \| SBP 120-129  & DBP 60-79 \| \| SBP 130-139  or DBP 80-89 \| \| SBP ≥140  or DBP ≥90 \| \| \| \| 18-29 (n=18,943) \| \|  \| \|  \| \|  \| \|  \| \|  \| \| \|  \| \| person-years \| \| 11,469.5 \| \| 36,770.2 \| \| 3,621.3 \| \| 4,169.8 \| \| 258.5 \| \| \|  \| \| Incident cases \| \| 146 \| \| 378 \| \| 30 \| \| 26 \| \| 5 \| \| \|  \| \| ID (per 10^3^ person-years) \| \| 12.7 \| \| 9.3 \| \| 5.8 \| \| 4.2 \| \| 8.1 \| \| \|  \| \| Multivariable HR (95% CI)^a^ \| \| 1.07 (0.89-1.30) \| \| reference \| \| 1.10 (0.75-1.59) \| \| 0.75 (0.50-1.12) \| \| 1.98 (0.82-4.78) \| \| \| 0.363 \| \| 30-39 (n=98,207) \| \|  \| \|  \| \|  \| \|  \| \|  \| \| \|  \| \| person-years \| \| 53,642.7 \| \| 235,158.7 \| \| 22,852.3 \| \| 51,188.7 \| \| 3,912.4 \| \| \|  \| \| Incident cases \| \| 538 \| \| 1,849 \| \| 144 \| \| 333 \| \| 23 \| \| \|  \| \| ID (per 10^3^ person-years) \| \| 10.0 \| \| 7.9 \| \| 6.3 \| \| 6.5 \| \| 5.9 \| \| \|  \| \| Multivariable HR(95% CI)^a^ \| \| 1.12 (1.02-1.24)* \| \| reference \| \| 0.93 (0.79-1.11) \| \| 0.94 (0.83-1.06) \| \| 0.88 (0.58-1.33) \| \| \| 0.013 \| \| 40-49 (n= 53,921) \| \|  \| \|  \| \|  \| \|  \| \|  \| \| \|  \| \| person-years \| \| 15,914.3 \| \| 131,423.7 \| \| 13,883.2 \| \| 45,523.6 \| \| 4,911.1 \| \| \|  \| \| Incident cases \| \| 134 \| \| 1,058 \| \| 94 \| \| 269 \| \| 22 \| \| \|  \| \| ID (per 10^3^ person-years) \| \| 8.4 \| \| 8.1 \| \| 6.8 \| \| 5.9 \| \| 4.5 \| \| \|  \| \| Multivariable HR (95% CI)^a^ \| \| 0.97 (0.81-1.16) \| \| reference \| \| 0.84 (0.68-1.04) \| \| 0.79 (0.69-0.90)* \| \| 0.64 (0.42-0.98)* \| \| \| <0.001 \| \| ≥50 (n=12,377) \| \|  \| \|  \| \|  \| \|  \| \|  \| \| \|  \| \| person-years \| \| 2,446.2 \| \| 22,685.0 \| \| 2,836.1 \| \| 8,751.7 \| \| 1,184.5 \| \| \|  \| \| Incident cases \| \| 14 \| \| 98 \| \| 17 \| \| 41 \| \| 3 \| \| \|  \| \| ID (per 10^3^ person-years) \| \| 5.7 \| \| 4.3 \| \| 6.0 \| \| 4.7 \| \| 2.5 \| \| \|  \| \| Multivariable HR (95% CI)^a^ \| \| 1.11 (0.63-1.94) \| \| reference \| \| 1.29 (0.77-2.17) \| \| 1.18 (0.82-1.70) \| \| 0.65 (0.21-2.05) \| \| \| 0.702 \|   Abbreviations: BP, blood pressure; CI, confidence interval; DBP, diastolic blood pressure; HR, hazard ratio; ID, incidence density; SBP, systolic blood pressure.  Note: P = 0.213 for the overall interaction between age and BP category for incident case-level depressive symptoms  ^a^ Estimated from parametric proportional hazard model. Multivariable model was adjusted for age, sex, center, year of screening exam, BMI, smoking status, alcohol intake, physical activity, educational level, total calorie intake, history of diabetes, and baseline CESD.  *Statistically significant differences (p<0.05).  **Supplementary Table 5.** Baseline characteristics according to CESD categories | | | | | |
| --- | --- | --- | --- | --- | --- | --- | --- | --- | --- | --- | --- | --- | --- | --- | --- | --- | --- | --- | --- | --- | --- | --- | --- | --- | --- | --- | --- | --- | --- | --- | --- | --- | --- | --- | --- | --- | --- | --- | --- | --- | --- | --- | --- | --- | --- | --- | --- | --- | --- | --- | --- | --- | --- | --- | --- | --- | --- | --- | --- | --- | --- | --- | --- | --- | --- | --- | --- | --- | --- | --- | --- | --- | --- | --- | --- | --- | --- | --- | --- | --- | --- | --- | --- | --- | --- | --- | --- | --- | --- | --- | --- | --- | --- | --- | --- | --- | --- | --- | --- | --- | --- | --- | --- | --- | --- | --- | --- | --- | --- | --- | --- | --- | --- | --- | --- | --- | --- | --- | --- | --- | --- | --- | --- | --- | --- | --- | --- | --- | --- | --- | --- | --- | --- | --- | --- | --- | --- | --- | --- | --- | --- | --- | --- | --- | --- | --- | --- | --- | --- | --- | --- | --- | --- | --- | --- | --- | --- | --- | --- | --- | --- | --- | --- | --- | --- | --- | --- | --- | --- | --- | --- | --- | --- | --- | --- | --- | --- | --- | --- | --- | --- | --- | --- | --- | --- | --- | --- | --- | --- | --- | --- | --- | --- | --- | --- | --- | --- | --- | --- | --- | --- | --- | --- | --- | --- | --- | --- | --- | --- | --- | --- | --- | --- | --- | --- | --- | --- | --- | --- | --- | --- | --- | --- | --- | --- | --- | --- | --- | --- | --- | --- | --- | --- | --- | --- | --- | --- | --- | --- | --- | --- | --- | --- | --- | --- | --- | --- | --- | --- | --- | --- | --- | --- | --- | --- | --- | --- | --- | --- | --- | --- | --- | --- | --- | --- | --- | --- | --- | --- | --- | --- | --- | --- | --- | --- | --- | --- | --- | --- | --- | --- | --- | --- | --- | --- | --- | --- | --- | --- | --- | --- | --- | --- | --- | --- | --- | --- | --- | --- | --- | --- | --- | --- | --- | --- | --- | --- | --- | --- | --- | --- | --- | --- | --- | --- | --- | --- | --- | --- | --- | --- | --- | --- | --- | --- | --- | --- | --- | --- | --- | --- | --- | --- | --- | --- | --- | --- | --- | --- | --- | --- | --- | --- | --- | --- | --- | --- | --- | --- | --- | --- | --- | --- | --- | --- | --- | --- | --- | --- | --- | --- | --- | --- | --- | --- | --- | --- | --- | --- | --- | --- | --- | --- | --- | --- | --- | --- | --- | --- | --- | --- | --- | --- | --- | --- | --- | --- | --- | --- | --- | --- | --- | --- | --- | --- | --- | --- | --- | --- | --- | --- | --- | --- | --- | --- | --- | --- | --- | --- | --- | --- | --- | --- | --- | --- | --- | --- | --- | --- | --- | --- | --- | --- | --- | --- | --- | --- | --- | --- | --- | --- | --- | --- | --- | --- | --- | --- | --- | --- | --- | --- | --- | --- | --- | --- | --- | --- | --- | --- | --- | --- | --- | --- | --- | --- | --- | --- | --- | --- | --- | --- | --- | --- | --- | --- | --- | --- | --- | --- | --- | --- | --- | --- | --- | --- | --- | --- | --- | --- | --- | --- | --- | --- | --- | --- | --- | --- | --- | --- | --- | --- | --- | --- | --- | --- | --- | --- | --- | --- | --- | --- | --- | --- | --- | --- | --- | --- | --- | --- | --- | --- | --- | --- | --- | --- | --- | --- | --- | --- | --- | --- | --- | --- | --- | --- | --- | --- | --- | --- | --- | --- | --- | --- | --- | --- | --- | --- | --- | --- | --- | --- | --- | --- | --- | --- | --- | --- | --- | --- | --- | --- | --- | --- | --- | --- | --- | --- | --- | --- | --- | --- | --- | --- | --- | --- | --- | --- | --- | --- | --- | --- | --- | --- | --- | --- | --- | --- | --- | --- | --- | --- | --- | --- | --- | --- | --- | --- | --- | --- | --- | --- | --- | --- | --- | --- | --- | --- | --- | --- | --- | --- | --- | --- | --- | --- | --- | --- | --- | --- | --- | --- | --- | --- | --- | --- | --- | --- | --- | --- | --- | --- | --- | --- | --- | --- | --- | --- | --- | --- | --- | --- | --- | --- | --- | --- | --- | --- | --- | --- | --- | --- | --- | --- | --- | --- | --- | --- | --- | --- | --- | --- | --- | --- | --- | --- | --- | --- | --- | --- | --- | --- | --- | --- | --- | --- | --- | --- | --- | --- | --- | --- | --- | --- | --- | --- | --- | --- | --- | --- | --- | --- | --- | --- | --- | --- | --- | --- | --- | --- | --- | --- | --- | --- | --- | --- | --- | --- | --- | --- | --- | --- | --- | --- | --- | --- | --- | --- | --- | --- | --- | --- | --- | --- | --- | --- | --- | --- | --- | --- | --- | --- | --- | --- | --- | --- | --- | --- | --- | --- | --- | --- | --- | --- | --- | --- | --- | --- | --- | --- | --- | --- | --- | --- | --- | --- | --- | --- | --- | --- | --- | --- | --- | --- | --- |
| Characteristics | Overall | CESD categories | | | *p* for trend |
|  |  | <16 | 16-24 | ≥25 |  |
| Naming of CESD categories |  | No depressive symptoms | Mild to moderate depressive symptoms | Severe depressive symptoms |  |
| Number | 172,102 | 152,720 | 13,480 | 5,902 |  |
| Age, years^a^ | 37.5 (7.2) | 37.7 (7.1) | 36.6 (7.3) | 36.0 (7.2) | <0.001 |
| Male (%) | 49.3 | 51.5 | 34.6 | 25.2 | <0.001 |
| Current smoker (%) | 24.8 | 25.2 | 22.3 | 19.0 | <0.001 |
| Alcohol intake (%)^c^ | 19.0 | 19.0 | 18.9 | 17.7 | 0.027 |
| HEPA (%) | 15.0 | 15.1 | 13.6 | 14.1 | <0.001 |
| High education level (%)^d^ | 85.1 | 85.9 | 79.4 | 76.7 | <0.001 |
| Diabetes (%) | 2.0 | 2.0 | 1.8 | 1.6 | 0.010 |
| Obesity (%)^e^ | 21.7 | 22.1 | 18.9 | 16.9 | <0.001 |
| Body mass index (kg/m^2^) | 22.7 (3.1) | 22.8 (3.1) | 22.4 (3.2) | 22.1 (3.3) | <0.001 |
| SBP | 104.6 (9.8) | 104.8 (9.8) | 102.8 (9.6) | 101.7 (9.4) | <0.001 |
| DBP | 66.3 (6.9) | 66.4 (6.9) | 65.4 (6.8) | 64.9 (6.8) | <0.001 |
| Glucose (mg/dl) ^a^ | 93.1 (11.5) | 93.2 (11.5) | 92.4 (11.7) | 91.9 (11.9) | <0.001 |
| Total cholesterol (mg/dl) ^a^ | 191.1 (33.0) | 191.4 (33.1) | 189.2 (32.3) | 187.4 (32.8) | <0.001 |
| LDL-C (mg/dl) ^a^ | 117.3 (31.0) | 117.8 (31.0) | 114.0 (30.4) | 111.7 (30.7) | <0.001 |
| HDL-C (mg/dl) ^a^ | 59.8 (15.1) | 59.6 (15.1) | 61.8 (15.5) | 62.7 (15.3) | <0.001 |
| Triglycerides (mg/dl)^†^ | 83 (60-121) | 84 (61-122) | 78 (58-113) | 74 (56-107) | <0.001 |
| hsCRP (mg/l) ^b^ | 1.11 (0.74-1.63) | 1.11 (0.74-1.63) | 1.10 (0.74-1.63) | 1.09 (0.73-1.62) | 0.181 |
| HOMA-IR ^b^ | 0.4 (0.2-0.8) | 0.4 (0.2-0.8) | 0.4 (0.2-0.8) | 0.3 (0.2-0.7) | <0.001 |
| Total energy intake (kcal/d)^b,f^ | 1,529.9 (1,173.0-1,919.5) | 1,533.1 (1,180.9-1,916.3) | 1,502.9 (1,116.0-1,941.3) | 1,506.6 (1,103.8-1,964.5) | <0.001 |
| Abbreviations: CESD, Center for Epidemiologic Studies-Depression scale; DBP, diastolic blood pressure; HDL-C, high-density lipoprotein-cholesterol; HEPA, health-enhancing physical activity; hsCRP, high sensitivity C-reactive protein; HOMA-IR, homeostasis model assessment of insulin resistance. LDL-C, low-density lipoprotein cholesterol; SBP, systolic blood pressure.  Data are expressed as ^a^mean (standard deviation), ^b^median (interquartile range), or percentage. ^c^≥ 20 g of ethanol per day; ^d^≥ College graduate; ^e^ BMI ≥25kg/m^2^; ^f^ Among 129,927 participants with plausible estimated energy intake levels (within three standard deviations from the log-transformed mean energy intake). | | | | | |

**Supplementary Table 6.** Development of case-level depressive symptoms by BP categories after excluding 807 subjects taking new anti-depressant use or 4,186 subjects taking new anti-hypertensive medication use during follow up

| BP categories |  | Multivariable-adjusted HR (95% CI)^a^ |
| --- | --- | --- |
| SBP <90 or DBP <60 | Hypotension | 1.08 (0.99-1.17) |
| SBP 90-119 & DBP 60-79 | Normal BP | 1.00 (reference) |
| SBP 120-129 & DBP 60-79 | Elevated BP | 0.95 (0.84-1.08) |
| SBP 130-139 or DBP 80-89 | Hypertension stage 1 | 0.90 (0.82-0.99) |
| SBP ≥140 or DBP ≥90 | Hypertension stage 2 | 0.89 (0.63-1.24) |
| *p* for trend |  | 0.002 |

Abbreviations: BP, blood pressure; CESD, Center for Epidemiologic Studies-Depression scale; CI, confidence intervals; DBP, diastolic blood pressure; HR, hazard ratio; SBP, systolic blood pressure.

a Estimated from parametric proportional hazard model. Multivariable model was adjusted for age, sex, center, year of screening exam, BMI, smoking status, alcohol intake, physical activity, educational level, total calorie intake, history of diabetes and baseline CESD

**Supplementary Table 7.** Development of hypertension by CESD categories after excluding 955 subjects taking new anti-depressant use or 1,116 subjects taking new anti-hypertensive medication use during follow up

| CESD categories | Naming of CESD categories | Multivariable-adjusted HR (95% CI)^a^ |
| --- | --- | --- |
| <16 | No depressive symptoms | 1.00 (reference) |
| 16-24 | Mild to moderate depressive symptoms | 1.06 (1.01-1.11) |
| ≥25 | Severe depressive symptoms | 1.10 (1.02-1.19) |
| *p* for trend |  | 0.002 |
| *Per 5 score increase in CESD* | | 1.01 (1.001-1.02) |

Abbreviations: BP, blood pressure; CI, confidence interval; DBP, diastolic blood pressure; HR, hazard ratio; SBP, systolic blood pressure.

^a^Estimated from parametric proportional hazard model. Multivariable model was adjusted for age, sex, center, year of screening exam, BMI, smoking status, alcohol intake, physical activity, educational level, total calorie intake, history of diabetes, and baseline CESD.

**Supplementary Table 8.** Baseline characteristics according to follow up loss

| Characteristics | Follow up | Follow up loss | *p* value | SMD |
| --- | --- | --- | --- | --- |
| Number | 276,244 | 141,634 |  |  |
| Age, years | 38.9 (8.2) | 43.0 (12.9) | <0.001 | 0.379 |
| Male (%) | 55.3 | 47.5 | <0.001 | 0.156 |
| Current smoker (%) | 28.1 | 24.2 | <0.001 | 0.089 |
| Alcohol intake (%)^a^ | 23.4 | 24.4 | <0.001 | 0.229 |
| HEPA (%) | 16.0 | 18.4 | <0.001 | 0.063 |
| High education level (%)^b^ | 83.2 | 67.6 | <0.001 | 0.367 |
| Hypertension (%)^c^ | 23.2 | 27.2 | <0.001 | 0.094 |
| Diabetes (%) | 3.7 | 6.7 | <0.001 | 0.135 |
| Obesity (%)^d^ | 27.7 | 28.7 | <0.001 | 0.024 |
| Body mass index (kg/m^2^) | 23.2 (3.3) | 23.4 (3.4) | <0.001 | 0.036 |
| SBP | 109.3 (13.1) | 110.2 (13.6) | <0.001 | 0.065 |
| DBP | 70.1 (10.0) | 70.3 (9.9) | <0.001 | 0.023 |
| Glucose (mg/dl) | 95.1 (14.2) | 96.4 (17.7) | <0.001 | 0.078 |
| Total cholesterol (mg/dl) | 193.7 (34.1) | 194.1 (35.5) | <0.001 | 0.012 |
| LDL-C (mg/dl) | 119.7 (31.9) | 121.7 (33.4) | <0.001 | 0.060 |
| HDL-C (mg/dl) | 58.5 (15.1) | 59.4 (15.8) | <0.001 | 0.057 |
| CESD ^e^ | 5.9 (2.4) | 6.7 (2.5) | <0.001 | 0.302 |

Abbreviations: CESD, Center for Epidemiologic Studies-Depression; DBP, diastolic blood pressure; HDL-C, high-density lipoprotein-cholesterol; HEPA, health-enhancing physical activity; hsCRP, high sensitivity C-reactive protein; HOMA-IR, homeostasis model assessment of insulin resistance; LDL-C, low-density lipoprotein cholesterol; SBP, systolic blood pressure; and SMD, standardized mean difference.

^a^≥ 20 g of ethanol per day; ^b^≥ College graduate; ^c^SBP ≥130 or DBP ≥80; ^d^ Body mass index ≥25kg/m^2^

Data are expressed as mean (standard deviation), ^e^ geometric mean (standard deviation), or percentage.

**Supplementary Table 9.** Development of case-level depressive symptoms by BP categories using inverse probability weights.

| BP categories |  | Multivariable-adjusted HR (95% CI)^a^ |
| --- | --- | --- |
| SBP <90 or DBP <60 | Hypotension | 1.09 (1.00-1.20) |
| SBP 90-119 & DBP 60-79 | Normal BP | 1.00 (reference) |
| SBP 120-129 & DBP 60-79 | Elevated BP | 0.98 (0.85-1.13) |
| SBP 130-139 or DBP 80-89 | Hypertension stage 1 | 0.88 (0.79-0.97) |
| SBP ≥140 or DBP ≥90 | Hypertension stage 2 | 0.60 (0.44-0.82) |
| *p* for trend |  | <0.001 |

Abbreviations: BP, blood pressure; CESD, Center for Epidemiologic Studies-Depression; CI, confidence interval; DBP, diastolic blood pressure; HR, hazard ratio; SBP, systolic blood pressure.

^a^ Estimated from parametric proportional hazard model *using inverse probability weights*. Multivariable model was adjusted for age, sex, center, year of screening exam, BMI, smoking status, alcohol intake, physical activity, educational level, total calorie intake, history of diabetes and baseline CESD.

To account for potential differences between participants with follow-up visits and those with no follow-up visit, we estimated the probability of having at least one follow-up visit as a function of baseline characteristics. Each individual was weighted by the inverse of the predicted probability of selection (inverse probability weight analysis [IPW]) to address the question “what would be the association between exposure and endpoint if there was no selection bias from the baseline study sample.”

**Supplementary Table 10.** Development of hypertension by CESD categories using inverse probability weights.

| CESD categories | Naming of CESD categories | Multivariable-adjusted HR  (95% CI)^a^ |
| --- | --- | --- |
| <16 | No depressive symptoms | 1.00 (reference) |
| 16-24 | Mild to moderate depressive symptoms | 1.05 (0.99-1.11) |
| ≥25 | Case-level depressive symptoms | 1.16 (1.06-1.29) |
| *p* for trend |  | 0.001 |
| *Per 5 score increase in CESD* | | 1.02 (1.01-1.03) |

Abbreviations: CESD, Center for Epidemiologic Studies-Depression; CI, confidence interval; DBP, diastolic blood pressure; HR, hazard ratio; SBP, systolic blood pressure.

^a^ Estimated from parametric proportional hazard model using inverse probability weights. Multivariable model was adjusted for age, sex, center, year of screening exam, BMI, smoking status, alcohol intake, physical activity, educational level, total calorie intake, history of diabetes and baseline SBP.

To account for potential differences between participants with follow-up visits and those with no follow-up visit, we estimated the probability of having at least one follow-up visit as a function of baseline characteristics. Each individual was weighted by the inverse of the predicted probability of selection (inverse probability weight analysis [IPW]) to address the question “what would be the association between exposure and endpoint if there was no selection bias from the baseline study sample.”

**Supplementary Table 11.** Baseline characteristics according to exclusion in the model for development of case-level depressive symptoms by BP category

| Characteristics | Those who are included in the analysis | Those who are excluded from the analysis due to missing values | *p* value | SMD |
| --- | --- | --- | --- | --- |
| Number | 183,448 | 37,674 |  |  |
| Age, years | 38.2 (7.3) | 39.0 | <0.001 | 0.105 |
| Male (%) | 57.0 | 50.6 | <0.001 | 0.364 |
| Current smoker (%) | 27.7 | 31.3 | <0.001 | 0.244 |
| Alcohol intake (%)^a^ | 22.3 | 22.9 | 0.007 | 0.055 |
| HEPA (%) | 15.3 | 17.3 | <0.001 | 0.208 |
| High education level (%)^b^ | 85.6 | 74.1 | <0.001 | 1.062 |
| Hypertension (%)^c^ | 16.8 | 19.1 | <0.001 | 0.229 |
| Diabetes (%) | 2.6 | 3.2 | <0.001 | 0.328 |
| Obesity (%)^d^ | 26.3 | 24.8 | <0.001 | 0.110 |
| Body mass index (kg/m^2^) | 23.1 (3.2) | 23.0 (3.2) | <0.001 | 0.057 |
| SBP | 108.3 (12.5) | 110.0 (12.8) | <0.001 | 0.134 |
| DBP | 69.5 (9.6) | 69.7 (9.4) | <0.001 | 0.025 |
| Glucose (mg/dl) | 94.4 (12.8) | 95.0 (14.0) | <0.001 | 0.044 |
| Total cholesterol (mg/dl) | 193.8 (33.9) | 193.2 (34.1) | 0.001 | 0.018 |
| LDL-C (mg/dl) | 120.1 (31.7) | 118.6 (31.6) | <0.001 | 0.050 |
| HDL-C (mg/dl) | 58.6 (15.0) | 59.0 (14.9) | <0.001 | 0.025 |
| CESD ^e^ | 4.8 (2.2) | 5.1 (2.1) | <0.001 | 0153 |

Abbreviations: SBP, systolic blood pressure; DBP, diastolic blood pressure; HDL-C, high-density lipoprotein-cholesterol; HEPA, health-enhancing physical activity; hsCRP, high sensitivity C-reactive protein; HOMA-IR, homeostasis model assessment of insulin resistance; LDL-C, low-density lipoprotein cholesterol; SBP, systolic blood pressure; SMD standardized mean difference.

^a^≥ 20 g of ethanol per day; ^b^≥ College graduate; ^c^ BMI ≥25kg/m^2^

Data are expressed as mean (standard deviation), ^d^ geometric mean (standard deviation), or percentage.

**Supplementary Table 12.** Baseline characteristics according to exclusion in the model for incident hypertension by CESD category

| Characteristics | Those who are included in the analysis | Those who are excluded from the analysis due to missing values | *p* value | SMD |
| --- | --- | --- | --- | --- |
| Number | 172,102 | 30,659 |  |  |
| Age, years | 37.5 (7.2) | 38.6 (8.2) | <0.001 | 0.143 |
| Male (%) | 49.3 | 44.7 | <0.001 | 0.259 |
| Current smoker (%) | 24.8 | 28.6 | <0.001 | 0.279 |
| Alcohol intake (%)^a^ | 19.0 | 19.7 | 0.003 | 0.069 |
| HEPA (%) | 15.0 | 16.7 | <0.001 | 0.186 |
| High education level (%)^b^ | 85.1 | 75.1 | <0.001 | 0.915 |
| Diabetes (%) | 2.0 | 2.6 | <0.001 | 0.439 |
| Obesity (%)^c^ | 21.7 | 20.4 | <0.001 | 0.105 |
| Body mass index (kg/m^2^) | 22.7 (3.1) | 22.6 (3.0) | <0.001 | 0.051 |
| SBP | 104.6 (9.8) | 106.0 (9.9) | <0.001 | 0.151 |
| DBP | 66.3 (6.9) | 66.4 (6.6) | 0.075 | 0.011 |
| Glucose (mg/dl) | 93.1 (11.5) | 93.8 (12.9) | <0.001 | 0.058 |
| Uric acid (mg/dl) | 5.1 (1.4) | 4.9 (1.4) | <0.001 | 0.123 |
| Total cholesterol (mg/dl) | 191.1 (33.0) | 191.0 (33.5) | 0.826 | 0.001 |
| LDL-C (mg/dl) | 117.3 (31.0) | 116.5 (31.2) | <0.001 | 0.027 |
| HDL-C (mg/dl) | 59.8 (15.1) | 59.9 (15.0) | 0.226 | 0.008 |
| CESD^d^ | 5.9 (2.4) | 6.0 (2.4) | 0.466 | 0.064 |

Abbreviations: SBP, systolic blood pressure; DBP, diastolic blood pressure; HDL-C, high-density lipoprotein-cholesterol; HEPA, health-enhancing physical activity; hsCRP, high sensitivity C-reactive protein; HOMA-IR, homeostasis model assessment of insulin resistance; LDL-C, low-density lipoprotein cholesterol; SBP, systolic blood pressure; SMD, standardized mean difference.

^a^≥ 20 g of ethanol per day; ^b^≥ College graduate; ^c^ BMI ≥25kg/m^2^

Data are expressed as mean (standard deviation), ^d^ geometric mean (standard deviation), or percentage.

**Supplementary Table 13.** Development of case-level depressive symptoms by BP category using missing value analysis.

| BP categories |  | Multivariable-adjusted HR (95% CI)^a^ |
| --- | --- | --- |
| SBP <90 or DBP <60 | Hypotension | 1.08 (1.01-1.15) |
| SBP 90-119 & DBP 60-79 | Normal BP | 1.00 (reference) |
| SBP 120-129 & DBP 60-79 | Elevated BP | 0.95 (0.85-1.05) |
| SBP 130-139 or DBP 80-89 | Hypertension stage 1 | 0.90 (0.83-0.96) |
| SBP ≥140 or DBP ≥90 | Hypertension stage 2 | 0.89 (0.72-1.11) |
| *p* for trend |  | <0.001 |

Abbreviations: BP, blood pressure; CI, confidence interval; DBP, diastolic blood pressure; HR, hazard ratio; SBP, systolic blood pressure.

^a^Estimated from Cox’s proportional hazard model using missing value analysis. Multivariable model was adjusted for age, sex, center, year of screening exam, BMI, smoking status, alcohol intake, physical activity, educational level, total calorie intake, history of diabetes, and baseline CESD.

**Supplementary Table 14.** Development of hypertension by CESD category using missing value analysis.

| CESD categories | Names of CESD categories | Multivariable-adjusted HR (95% CI)^a^ |
| --- | --- | --- |
| <16 | No depressive symptoms | 1.00 (reference) |
| 16-24 | Mild to moderate depressive symptoms | 1.06 (1.01-1.11) |
| ≥25 | Case-level depressive symptoms | 1.11 (1.03-1.20) |
| *p* for trend |  | <0.001 |
| *Per 5 score increase in CESD* | | 1.01 (1.003-1.02) |

Abbreviations: CESD, Center for Epidemiologic Studies-Depression; CI, confidence interval; DBP, diastolic blood pressure; HR, hazard ratio; SBP, systolic blood pressure.

^a^ Estimated from Cox’s proportional hazard model using missing value analysis*.*

Multivariable model was adjusted for age, sex, center, year of screening exam, BMI, smoking status, alcohol intake, physical activity, educational level, total calorie intake, history of diabetes and baseline SBP.
